# Blockage of neuromuscular glutamate receptors impairs reinnervation following nerve crush in adult mice

**DOI:** 10.3389/fncel.2022.1000218

**Published:** 2022-09-22

**Authors:** Kirkwood E. Personius, Danielle Siebert, Dennis W. Koch, Susan B. Udin

**Affiliations:** ^1^Program in Neuroscience, School of Medicine and Biomedical Sciences, University at Buffalo, Buffalo, NY, United States; ^2^Department of Rehabilitation Science, School of Public Health and Health Professions, University at Buffalo, Buffalo, NY, United States; ^3^Department of Kinesiology, Canisius College, Buffalo, NY, United States; ^4^Department of Physiology and Biophysics, School of Medicine and Biomedical Sciences, Buffalo, NY, United States

**Keywords:** glutamate, regeneration, pruning, polyneuronal, *N*-methyl-D-aspartate, neuromuscular junction

## Abstract

Motor axons in peripheral nerves are capable of regeneration following injury. However, complete recovery of motor function is rare, particularly when reinnervation is delayed. We have previously found that glutamate receptors play a crucial role in the successful innervation of muscle during mouse development. In particular, blocking N-methyl-D-aspartate (NMDA) receptor activity delays the normal elimination of excess innervation of each neuromuscular junction. Here, we use behavioral, immunohistochemical, electrophysiological, and calcium imaging methods to test whether glutamate receptors play a similar role in the transition from polyneuronal to mono-innervation and in recovery of function following peripheral nerve injury in mature muscle.

## Introduction

Peripheral nerve injury (PNI) is one of the most common causes of sensorimotor deficits and loss of productivity in adults. Injury to peripheral nerves disproportionately afflicts young healthy people and wounded soldiers, with large proportions demonstrating persistent weakness (Rivera et al., 2014; Huckhagel et al., 2018; Bergmeister et al., 2020). More than 20 million Americans are affected by PNI related ailments, with 3 of 100 admittances to trauma centers specific to nerve injury (Huckhagel et al., 2018). The muscle atrophy and chronic pain seen after PNI frequently requires physical and occupational services for more than 3 months, and 40% of these patients are unable to return to work 1 year post-injury (Padovano et al., 2020). Thus, failure of regenerated motor neurons to re-establish functional connections with muscle fibers after PNI is a significant clinical problem. Crushed or transected peripheral nerves can regrow, but motor neuron reinnervation often fails to restore normal function, especially if there is a long delay before the axons reach the muscles. In this paper, the possible contribution of neuromuscular N-methyl-D-aspartate (NMDA)-type glutamate receptors to recovery of normal connections patterns and function is evaluated.

A major impetus for this study stemmed from observations of developing neuromuscular connections: NMDA receptors contribute to the elimination of excess (polyneuronal) innervation (Personius et al., 2016). Polyneuronal innervation also occurs in adults when regenerating motor neurons regrow to the neuromuscular junction (NMJ) (Rich and Lichtman, 1989; Ribchester and Barry, 1994). NMDA receptors are found using immunohistochemistry to be localized at the neuromuscular junction in rodents (Berger et al., 1995). A major source of the glutamate that activates these receptors arises through the breakdown of the synaptically released precursor *N*-acetylaspartylglutamate (NAAG) into glutamate and *N*-acetylasparate; this breakdown is mediated by the enzyme GCPII that is expressed by terminal Schwann cells (Berger et al., 1995; Marmiroli et al., 2012). The NMDA receptors contribute rises in calcium concentration in the muscle, notably at the neuromuscular junction (Personius et al., 2016). This local increase in calcium may help to activate nitric oxide synthase, which is localized to the same neuromuscular junction region as the NMDA receptors (Berger et al., 1995; Urazaev et al., 1995; Malomouzh et al., 2005). Nitric oxide, which can diffuse across the synapse to the presynaptic terminals, is likely to act as a retrograde messenger (Grozdanovic and Gossrau, 1998). There is evidence that nitric oxide may mediate the glutamate-induced reduction in non-quantal acetylcholine release (Malomouzh et al., 2003), and influence synapse elimination during development (Vyskocil and Vrbova, 1993).

This paper documents the contribution of NMDA receptors in promoting the return to single innervation and in restoration of function during the first weeks after nerve crush. The expression of NMDA receptors at the endplate after regeneration was confirmed by immunostaining and calcium imaging. To test the functional impact of the NMDA receptors, we immunostained the regenerating neuromuscular connections following sciatic nerve crush and observed that blockade of glutamate receptors resulted in prolonged retention of polyneuronal innervation and abnormal patterns of axon growth at the NMJ. Similarly, the toe-spread behavioral test demonstrated that glutamate receptors signaling was required for the normal rate of restoration of function. Electrophysiological testing showed a greater resistance to neuromuscular fatigue during a nerve-fatigue protocol when NMDA receptors were blocked.

## Materials and methods

### Animals

Experiments were performed in adult CD-1 mice of either sex (Charles RIver Laboratories, Wilmington, MA, USA). Procedures were approved by the Institutional Animal Care and Use Committee of the University at Buffalo. Animals were euthanized by intraperitoneal injection of ketamine/xylazine (100/10 mg/kg).

### Elvax implants

For slow release of drugs, Elvax 40W (Ethylene vinyl acetate, gift of Dupont, Wilmington, DE, USA) polymer was impregnated with D-2-amino-5-phosphonopentanoate (D-AP5) (100 μM) (catalog #0106; R&D Systems, Inc., Minneapolis, MN, USA) and CNQX (100 μM; catalog #104510; ThermoFisher Scientific, Waltham, MA, USA), or saline. Sheets of the material were prepared as previously described (Personius et al., 2016). To implant the Elvax, mice were anesthetized with isoflurane. A small incision was made in the lateral skin of the distal hindlimb, and the fascial plane between the anterior and posterior compartments was cut. A 4–5 mm rectangle of Elvax (10 mg) was placed bilaterally between the two compartments. The incision was sutured and mice were returned to the cage after warming. Drug-impregnated or saline-impregnated Elvax was implanted at the time of nerve crush and left in place for up to 63 days.

### Nerve crush

The right sciatic nerve was crushed 3 mm proximal to nerve trifurcation following the protocol of Bauder and Ferguson (2012) to improve surgical reproducibility between animals. The sciatic nerve was crushed twice at the same site using super-fine hemostatic forceps (Fine Science Tools (USA), Inc., Foster City, CA, USA, 13020-12). The crush was held for 15 s at 3 clicks of the hemostatic forceps. The crush site was marked with sterilized powdered carbon. All animals with sciatic nerve crush underwent Elvax implantation. Elvax impregnated with AP5 and CNQX was implanted in the experimental group (Drug-crush). Elvax impregnated with saline was implanted in the control group (Saline-crush).

### Calcium imaging

#### Solutions and drugs

A magnesium-free Ringer’s medium was used for all experiments in order to avoid channel-block of NMDA receptors. It contained the following (in mM): 25 NaHCO_3_, 2 CaCl_2_, 11 glucose, 0.4 glutamine, 15 BES [*N*, *N*-bis(2-hydroxyethyl)-2-aminoethanesulfonic acid], 113 NaCl, 2 KCl, 0.01 glycine, 0.036 choline chloride, 4.34 × 10^–7^ cocarboxylase [thiamine pyrophosphate], 3 lidocaine, bubbled in 95% O_2_/5% CO_2_; 0.1 mM atropine and 0.004 μM tubocurarine were included to minimize activation of cholinergic receptors. Muscles were incubated for 30 min in Ringer’s medium with 10 g/ml α-rhodamine-bungarotoxin (which served both to enable visualization of the end plate and to block cholinergic receptors), 1 μl/ml Pluronic F-127 (20% solution in DMSO, catalog #P-3000MP, Life Technologies, used to disperse the acetoxymethyl esters of Fluo-4), and 10 μl/ml Fluo-4 AM (1 mM solution in DMSO, catalog #F-14217; Life Technologies, Grand Island, NY, USA) and then rinsed for a minimum of 20 min in Ringer’s medium.

#### Imaging

Experiments were performed at 20–24°C. The soleus or extensor digitorum longus (EDL) muscles were placed in a perfusion chamber (Warner Instruments, Holliston, MA, USA) with a 230 μl working bath volume and perfused at a rate of 2 ml/min. Muscles from the limb opposite to the nerve crush were used for comparison (“non-crush”). Muscles from animals with no crush were used as controls and the resulting data are denoted “naïve.” Excitation light was provided by a 103 W/2 mercury short arc lamp (Olympus, Westborough, MA, USA) and was attenuated by using neutral density filters to minimize photobleaching. Images were collected with an Olympus BX51WI microscope equipped for epifluorescence using an FITC filter (excitation wavelength 490, emission 520) and an Olympus 40 × (numerical aperture 0.8) water-immersion lens using a Cooke Sensicam QE. Exposure times were 400 ms, and image frames were taken every 3 s. Acquisitions were performed with SLIDEBOOK software (Intelligent Imaging Innovations, Inc., Denver, CO, USA). For each field of endplates, the following sequence of captures was followed: control, control, NMDA, control, and 40 mM KCl. Thus, the time course was 60 s of saline perfusion, 60 s of saline perfusion, 60 s of NMDA perfusion, 5 min of saline wash, and 60 s of KCl perfusion. Only muscles that showed obvious responses to NMDA and/or visibly responded to 40 mM KCl were used for analysis. The attached nerve was not intentionally stimulated.

#### Image analysis

Image analysis was performed in Fiji (ImageJ). For measurement of changes in Ca^+2^ response, a region of interest over the acetylcholine receptor (AChR) region was identified as visualized by rhodamine ∝-bungarotoxin. Changes of calcium levels are presented as normalized Δ*F/F*, where *F* is the resting fluorescence (before stimulation) and Δ*F* is the peak change in fluorescence from resting levels. Fluorescence values were assessed after normalizing by subtracting fluorescence intensity in the region of interest over the AChRs from fluorescence intensity in adjacent control areas of the same size.

### Immunohistochemistry

To visualize NMDA receptor expression, muscles were immersion-fixed for 15 min in 4% paraformaldehyde at pH 7.4, rinsed 3 times, embedded in OCT compound, frozen in isopentane cooled by liquid nitrogen, sectioned at 20 μm on a cryostat and mounted on microscope slides. The tissue was rinsed 3× in phosphate-buffered saline (PBS) followed by 20 min in 10% Alexa 488-conjugated-∝-bungarotoxin (catalog B13422, ThermoFisher) and 3 more rinses. The muscles were blocked for 50 min in saline with 2% bovine serum albumin, 0.1% sodium azide and 1% Tween followed by 1 rinse and incubation overnight at room temperature with rabbit primary antibody to the gluN1 subunit of the NMDA receptor (Invitrogen PA3-102, 1:500). The next day, following 3 rinses in blocker, Alexa Fluor Goat anti-Rabbit 555 (1:500, Invitrogen A32732) and DyLight 405 Goat anti-Mouse (1:250, Novus NBP1-72886) were applied for 2 h. Staining was visualized by conventional epifluorescence microscopy (63× oil objective, BX51; Olympus) and confocal microscopy (63× oil objective, LSM 510 Meta NLO; Zeiss Microscopes, Feasterville, PA, USA).

### Analysis of multiple innervation

The soleus and EDL muscles were pinned in saline in a Sylgard-coated dish and cleaned of connective tissue. They were immersion-fixed for 15 min in 4% paraformaldehyde at pH 7.4, placed in microfuge tubes and rinsed 3× in PBS, followed by 20 min in 10% rhodamine-conjugated-∝-bungarotoxin (catalog T1175, Life Technologies, Grand Island, NY, USA) and 3 more PBS rinses. After immersion for 5 min in −20° methanol, the muscles were rinsed 3X in PBS and blocked for 60 min in saline with 2% bovine serum albumin, 0.1% sodium azide and 0.2% Triton-X100. The muscles were bathed overnight at room temperature in primary antibodies to SV2 (synaptic vesicles, 1:20, RRID:AB_2315390, Developmental Studies Hybridoma Bank, Iowa City, IA, USA) and 2H3 (neurofilament, 1:10, RRID:AB_531793; Developmental Studies Hybridoma Bank) and visualized by secondary antibody Alexa Fluor 488 donkey anti-mouse (RRID:AB_2556542, catalog #R37114; Life Technologies, Grand Island, NY, USA). Slides were coverslipped with Vectashield mounting medium. Staining was visualized by conventional epifluorescence microscopy (63× oil objective, BX51; Olympus) and confocal microscopy (63× oil objective, LSM 510 Meta NLO; Zeiss). The number of axons innervating each end plate was counted.

### Behavioral testing: Toe-spread test

The return of the toe spread reflex was assessed as a sensitive measure of restored function (Bozkurt et al., 2011). The degree of effective reinnervation of the anterior tibialis, EDL and intrinsic foot muscles was assessed at 2–20 days post-crush (10 mice with inhibitor-infused implants and 10 with saline-infused implants). Mice were gently covered with a piece of cloth and lifted by the tail to induce the hindleg digits to spread maximally. The reflex was scored as: 0, no spreading; 1, intermediate spreading of toes; and 2, full spreading (Bozkurt et al., 2011; Ma et al., 2011). See Figure 4.

**FIGURE 1:**
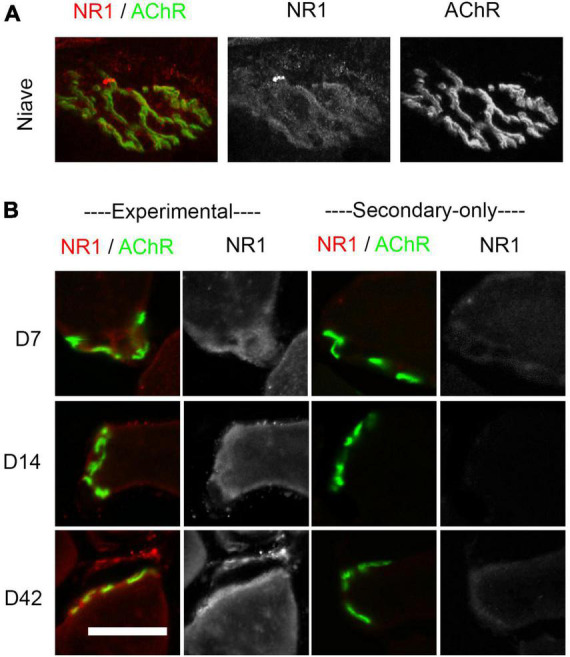
Immunostaining of NMDA receptors at the endplate. **(A)** Confocal images of whole-mount extensor digitorum longus (EDL) endplate showing overlap between NMDA-GluN1 subunits (red) and ∝-bungarotoxin-stained ACh receptors (green) from a non-injured adult mouse. Greyscale images of the same endplate are shown for GluN1 and AChR immunolabeling in the middle and right images, respectively. **(B)** Muscles from sectioned soleus or EDL muscles at 7–42 days after sciatic nerve crush. Column 1: NMDA-GluN1 subunits (red) and ∝-bungarotoxin-stained ACh receptors (green). Column 2: GluN1 subunit greyscale staining. Right: The 3rd and 4th columns show secondary-only controls at 7–42 days in which the primary antibody was omitted. Experimental and secondary-only images were taken on the same day using the same confocal settings. Scale bar = 50 μm.

**FIGURE 2:**
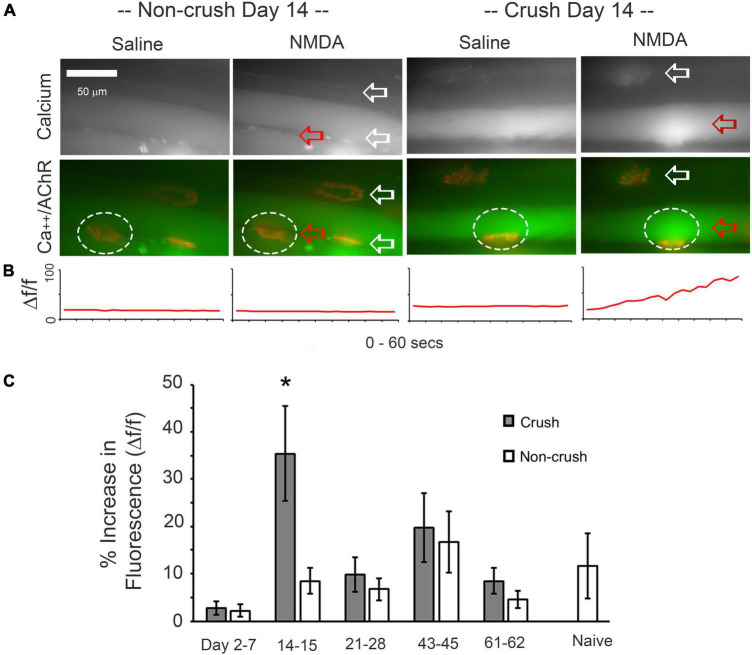
Responses of soleus or extensor digitorum longus (EDL) muscle fibers to bath-applied NMDA. **(A)** Comparison of fibers contralateral to the nerve-crush limb and fibers from muscles at 14 days after nerve crush. Top row shows fluorescence of Fluo-4, with arrows pointing to ∝-bungarotoxin labeling of endplates. Lower row shows merged channels illustrating the relationship of the rise in calcium to the location of endplates during a 30 s period of bath-perfused NMDA application, with ∝-bungarotoxin-labeled ACh receptors shown in red. **(B)** Normalized change fluorescence over the 60 s bath-applied period for saline or NMDA for the NMJ region of interest (oval) indicated by the red arrow. **(C)** Normalized increase in fluorescence in response to NMDA at 2–62 days after nerve crush and in naïve muscles. Non-crush data are taken from the muscles contralateral to the nerve crush. A significant increase in Fluo-4 fluorescence was only seen at 14–15 days post-sciatic nerve crush (asterisk, *p* < 0.05, two-way *t*-test, *n* = 7–20 muscles).

**FIGURE 3:**
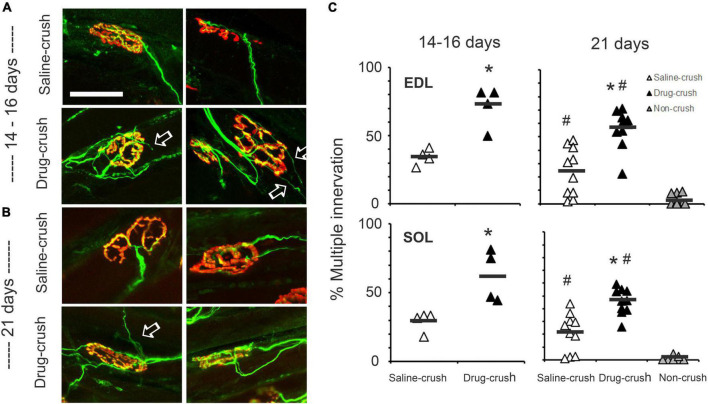
Elimination of polyneuronal innervation. **(A)** Examples of neuromuscular innervation in extensor digitorum longus (EDL) muscles at 14–16 days post-crush. Red: acetylcholine receptors at NMJ; green: regenerated sciatic nerve axons. The top row shows images from muscles treated with saline-impregnated Elvax (saline-crush), while the bottom row shows images from muscles treated with AP5&CNQX-impregnated Elvax (saline-crush). White arrows indicate examples of axon branches that extend beyond the endplate. **(B)** Neuromuscular junctions in EDL muscles at 21 days post-crush from muscles treated with saline-impregnated Elvax (saline-crush, top) or AP5&CNQX-impregnated Elvax (saline-crush, bottom). **(C)** Percentages of muscles with multiple innervation. Each symbol represents counts from a single muscle. Data from the EDL muscles at 14–16 or 21 days post-crush are illustrated in the upper row, while data from the soleus muscle are shown below. Open triangles: saline treatment; black triangles: AP5&CNQX-treated muscles. The extreme right columns in the 21-day charts also show the percentages of multiply-innervated axons from control muscles with no nerve crush (non-crush, gray triangles). Asterisks indicate difference from saline-crush; hashtags indicate difference at 21 days from non-crush control, *p* < 0.05, two-way *t*-test or one-way ANOVA Tukey *post-hoc* test. *n* = 4 for saline and drug-crush at 14–16 days. *n* = 10 for saline and drug-crush and 6 for non-crush at 21 days. Scale bar = 50 μm. ^#^Indicate difference at 21 days from non-crush control.

**FIGURE 4:**
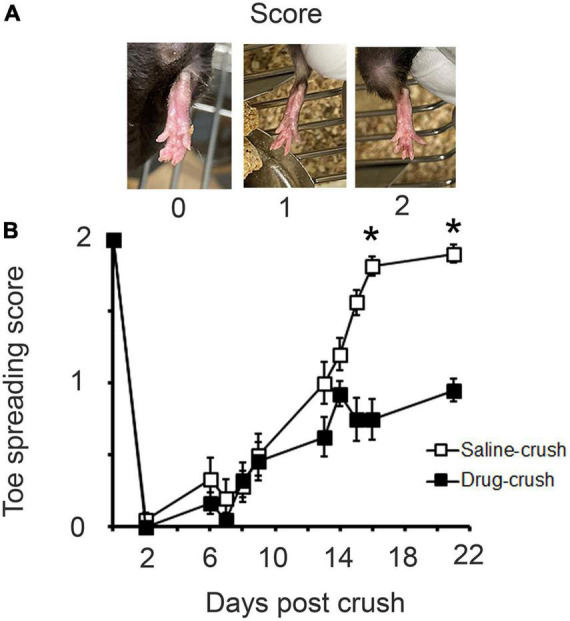
Recovery of toe-spread reflex. **(A)** Scoring scheme for quantification of recovery of toe spreading after nerve crush. **(B)** Comparison of recovery rates for saline-treated (open squares) vs. AP5&CNQX-treated (filled squares) animals. Asterisks indicate that a significant difference is reached by day 16. Non-parametric Friedman test, *p* < 0.001, *n* = 10 animals for saline and drug-crush.

### Muscle contractile properties and neurotransmission failure

The soleus muscle was transferred to a water jacket bath filled with constantly aerated Krebs solution at 27°C and fixed at the calcaneal tendon *via* alligator clip, while the proximal tendon was fixed to the arm of an isometric force transducer *via* suture (300B Series Lever System, Aurora Scientific, Inc., Aurora, ON, Canada). The muscle was directly stimulated by two chlorided silver plate electrodes using rectangular pulses of anodal current with 2.0 ms duration at supra-maximal voltage (Grass S88 stimulator with custom current amplifier, Astro-Med, Inc., West Warwick, RI, USA). Prior to determining maximal isometric muscle force, optimal muscle fiber length for force production (Lo) was obtained by determining the muscle length that produced the greatest twitch force. Isometric force/frequency data was acquired by measuring force output during administration of 900 ms pulse trains with intra-train pulse rates of 10, 20, 35, 50, 65, 80, 100, 125, 150, and 200 Hz.

Following a 5-min recovery period, the tibial nerve was then pulled into a suction electrode so that the muscle could be activated *via* indirect nerve stimulation using rectangular pulses of anodal current with 2.0 ms duration (Master 8, AMPI, Int., Jerusalem, Israel). The Neurotransmission failure (NF) protocol was carried-out at 80 Hz. NF was measured using methods previously described (Personius and Sawyer, 2006). The aim was to compare force production in response to direct trains of stimulation to force induced by stimulation of the tibial nerve. The force exerted due to direct stimulation is expected to decrease with time due to muscle fatigue, but the force exerted by the muscle when it is stimulated *via* the nerve is expected to decrease even more due to the progressive inability of the neuromuscular synapse to activate the muscle.

The soleus muscle was stimulated *via* the tibial nerve with 330-ms trains at 1 Hz with an intra-train pulse rate of 80 Hz. A single train of direct muscle stimulation with similar train characteristics was superimposed upon the nerve stimulation every 15 s. The alternating stimulation paradigm between nerve and direct muscle stimulation continued for 285 s. NF was determined every 15 s using the equation NF = (F–MF)/(1–MF) where NF is neurotransmission failure, F is percent force loss during nerve stimulation, and MF is percent force loss due to contractile fatigue (Kuei et al., 1990). Force measurements were recorded and analyzed using Spike2 V6.07 (Cambridge Electronic Design, Cambridge, England).

### Statistics

Neurotransmission failure and contraction force were analyzed by two-way ANOVA with Tukey *post-hoc* test. Toe spread was analyzed by a non-parametric Friedman test. Frequency of multiple-innervation vs. single-innervation was assessed by one-way ANOVA with Tukey *post-hoc* test or two-way *t*-test. Pre-NMDA vs. post-NMDA Ca^2^ imaging relative brightness was assessed by paired *t*-test. The data variances met the criteria for use of *t*-tests. Significance was defined as *p* ≤ 0.05.

## Results

### Immunostaining shows the presence of NMDA receptors at the endplate

To investigate the expression of NMDA receptors after nerve crush, the EDL and soleus muscles were stained with antibodies to the GluN1 subunit of the receptor at 7–42 days post-crush. Figure 1A illustrates the co-expression of these receptors with ∝-bungarotoxin-labeled ACh receptors at the endplate in a whole-mount preparation from a naive control muscle as previously reported (Berger et al., 1995; Mays et al., 2009). Figure 1B shows GluN1 labeling following sciatic nerve crush in muscle cross-sections (Experimental). We used cross-section preparations to increase the number of NMJs we could image per muscle. While the staining for NMDA receptors was co-extensive with AChRs, the labeling appeared to extend beyond the endplate region, particularly during the early stages of regeneration (D7 and D14). Omission of primary antibody (Secondary-only) results in no staining for NMDA receptors (Figure 1B, Secondary-only). Moreover, although we observed NMDA receptor immunostaining during regeneration of the crushed nerve, such staining was apparent only in a small subset of endplates. As previously reported, GluN1 labeling was also found at the NMJ in a subset of adult non-injured muscles (Figure 1B; Berger et al., 1995; Mays et al., 2009).

### Calcium imaging demonstrates a transient period of muscle responses to NMDA after nerve crush

We also assessed muscle NMDA receptor expression by using Fluo-4 to evaluate whether NMDA caused a rise in calcium levels in muscles at various times after nerve crush. Despite the anatomical presence of NMDA receptors at the NMJ from at least day 7 to day 42 post-crush as shown in Figure 1. The responses to NMDA began with a rise in calcium levels at the endplate region and often spread to adjacent regions of the muscle (Arrow in Figure 2A). The time-course of the change in relative fluorescence is illustrated in Figure 2B for the circles areas in Figure 2A. Figure 2C shows that 30 s of bath-applied NMDA induces significant increases in calcium levels only at 14–15 days post-crush, although NMDA-induced calcium influx was seen at a few individual NMJs at all time-points tested (*n* = 7–20 muscles per time-point).

### Blocking ionotropic glutamate receptors during regeneration slows elimination of polyneuronal innervation after nerve crush

After sciatic nerve crush, the regenerating nerve reaches the endplates of the soleus and EDL muscles and initially establishes multiple inputs to each motor endplate (Figure 3). To explore whether NMDA receptors influence the rate of elimination of this polyneuronal innervation, we blocked NMDA and α-amino-3-hydroxy-5-methyl-4-isoxazolepropionic acid (AMPA) receptors by implanting a sheet of the slow-release polymer Elvax infused with the blockers AP5 (100 mM) and CNQX (100 mM) at the time of the nerve crush; the implant was placed in the fascial plane between the anterior and posterior compartments of the distal hindlimb in order to allow the drugs to have access to the NMJs of the soleus and EDL muscles (Drug-crush). For controls, the same procedure was followed, but the Elvax was infused with saline rather than with receptor blockers (Saline-crush).

To assess the effect of the ionotropic glutamate receptor blockage on the elimination of polyneuronal innervation, we removed soleus and EDL muscles at 14–16 days post-crush. As Figure 3A shows, the saline-implant limbs showed substantial elimination of multiple endplates, as expected. In contrast, approximately twice as many muscles with the glutamate receptor inhibitors still had multiple innervation as compared with saline-implant controls (Day 14–16 EDL: 71.7 ± 7.5% drug vs. 34.4 ± 3.0% saline, *p* = 0.004 two-tailed *t*-test, *n* = 4, 4; Day 14–16 SOL 61.9 ± 9.4% drug vs. 29.1 ± 3.7% saline EDL, *p* = 0.03, two-tailed *t*-test, *n* = 4, 4, mean ± SEM). See Figure 3C, 14–15 days.

As shown in Figure 3B, significantly greater multiple innervation persisted at 21 days for both the EDL and the soleus muscles when glutamate receptors were blocked compared to saline-implant muscles (Day 21 EDL: 56.1 ± 4.5% drug vs. 24 ± 5.6% saline, *n* = 10, 10 Day 21 SOL 45.6 ± 3.2% drug vs. 21.2. ± 4.8% saline, *n* = 10, 10). Muscles in limbs contralateral to the nerve had significantly less multiple innervation than either the AP5&CNQX-block and saline-implant control muscles on the side of crush (non-crush EDL: 2.7 ± 1.3%, SOL 0.8 ± 0.7%, *n* = 6, one-way ANOVA with Tukey *post-hoc*, *p* = 0.001). See Figure 3C, 21 days.

To determine whether glutamate receptor blockade affected the number of axons innervating each muscle fiber endplate, we counted the number of innervating axons per endplate at 21 days post-crush. The EDL muscles of AP5&CNQX-block mice had 1.6 ± 0.05 inputs per muscle while saline-treated had only 1.2 ± 0.06 (*n* = 10, 10, *p* < 0.001 two-tailed *t*-test). The SOL muscle showed a similar increase (*n* = 10, 10, 1.5 ± 0.04 vs. 1.2 ± 0.05 inputs, *p* < 0.001 two-tailed *t*-test).

Regenerated axons in many of the muscles treated with glutamate inhibitors extended sprouts beyond the endplate that they had innervated (arrows, Figures 3A,B; Rich and Lichtman, 1989). This pattern was never observed in normal muscles or in control regenerated cases at these intervals after nerve crush. Thus, blocking glutamate receptors during regeneration not only slows recovery of normal reinnervation but also leads to unusual patterns of axonal growth, observations that are consistent with a role for NMDA and/or AMPA receptors in restoration of connectivity at the NMJ.

### Blocking ionotropic glutamate receptors during regeneration slows behavioral recovery after nerve crush

To test whether the effect of glutamate blockers on the anatomical rate of maturation at the endplate was paralleled by functional consequences, the toe-spread behavioral test was performed between 2 and 21 days post-crush (Figure 4). This test reflects the functional capacity of the EDL muscle, which is necessary for extension of the toes. The limbs with saline implants showed steady recovery beginning at about day 6 and plateauing at almost full recovery about day 13. In contrast, the limbs treated with AP5&CNQX (“Drug-crush”) showed a much slower rate of recovery and failed to recover fully. The saline-treated and AP5&CNQX-treated limbs’ scores were indistinguishable between days 6 and 9 and then began to diverge at around day 13, with lower scores for the drug-treated limbs, reaching statistical significance by day 16, *n* = 10 mice for Saline-crush and Drug-crush.

### Blocking ionotropic glutamate receptors during regeneration induces soleus atrophy and decreases neurotransmission failure, but does not alter total muscle contractile force

#### Muscle force and mass

**FIGURE 5:**
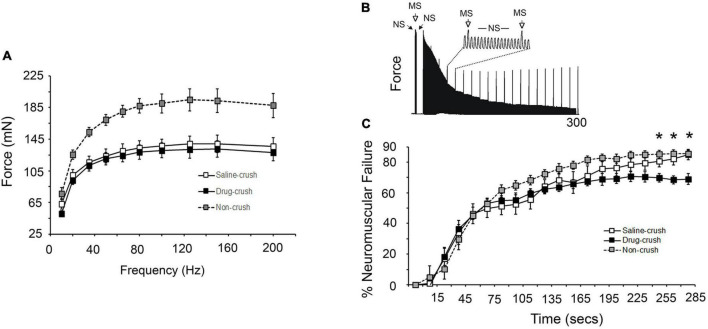
Electrophysiological assessment of soleus force production and neural failure. **(A)** Force generated by direct simulation of soleus muscles at different frequencies at 21 days post-crush. There is no significant difference between the saline-crush (open squares) and AP5&CNQX-treated (drug-crush) muscles (black squares). Gray squares show data from control muscles with no nerve crush. Control non-crush muscles were significantly stronger at all stimulation frequencies compared to saline-crush or drug-crush muscles (*n* = 4 muscle for saline and drug-crush and 6 for non-crush, two-way ANOVA with Tukey *post-hoc* test, *p* < 0.001). **(B)** Representative tracing of force measurements where direct muscle stimulation (MS) was intermittently superimposed every 15 s on trains of tibial nerve stimulation (NS). Difference in force production between MS and NS stimulation represents the contribution of neuromuscular transmission failure to muscle fatigue. **(C)** Increases in percent neuromuscular failure over 285 s of stimulation MS vs. NS stimulation. Neuromuscular failure was significantly lower in drug-crush soleus compared to saline-crush soleus by 255 s (asterisks, two-way ANOVA with Tukey *post-hoc* test, *p* < 0.001). NF was determined every 15 s using the equation NF = (F–MF)/(1–MF) where NF is neurotransmission failure, F is percent force loss during nerve stimulation, and MF is percent force loss due to contractile fatigue.

To determine whether NMDA receptors influence the recovery of muscle force, we removed the Elvax implants and then measured soleus contraction force at increasing stimulation frequencies at 21 days following nerve crush. Nerve crush resulted in a significant decrease in isometric contraction force compared to the opposite non-crushed limb at all stimulation frequencies (*n* = 4 muscles for saline and drug-crush and 6 for non-crush, 2-way ANOVA with Tukey *post-hoc* test, *p* < 0.001, Figure 5A). There was a trend for AP5&CNQX-blocked soleus muscles to be weaker than saline treated muscles on the side of crush, but significance was not reached (Figure 5A). Muscle mass (wet weight after blotting), however, was significantly reduced in the AP5&CNQX-blocked soleus on the side of crush compared to saline-treated and non-crushed soleus muscles (one-way ANOVA with Tukey *post-hoc* test; *p* = 0.032, Table 1). No differences were seen in optimal muscle length, twitch force, time to peak twitch force, or ½ relaxation time between groups (Table 1).

**TABLE 1:** Electrophysiological assessment of soleus mass, length, and force production.

	Muscle mass (mg)	Muscle length (l_o_ mm)	Twitch force (mN)	TTP (ms)	$\frac{1}{2}$ RT (ms)	NS/MS ratio (%)	*n*
Non-crush	8.6 ± 1.0	10.2 ± 0.5	76.5 ± 7.5	4.1 ± 0.2	7.1 ± 0.4	108 ± 4.1	5–8
Saline-crush	8.0 ± 0.1	0.94 ± 0.6	63.5 ± 3.8	4.5 ± 0.3	7.8 ± 0.4	94.1 ± 3.1	4
Drug-crush	6.5 ± 1.0*	10.1 ± 0.5	51.1 ± 6.5	3.7 ± 0.3	6.4 ± 0.6	93.7 ± 2.9	4
*p*-value	0.032	0.402	0.053	0.241	0.235	0.015	

Time to peak twitch force tension (TTP), $\frac{1}{2}$ relaxation time from peak twitch force (1/2 RT), ratio of initial force production following stimulation of the tibial nerve vs. direct muscle stimulation (NS/MS Ratio). Asterisk, one-way ANOVA with Tukey *post-hoc* test, *p* = 0.032.

#### Neuromuscular failure

Measurement of Neurotransmission Failure (NF) requires measurement of muscle force induced by direct soleus muscle stimulation compared to stimulation *via* the tibial nerve. Figure 5B illustrates the protocol for the experiment shown in Figure 5C. Repetitive stimulation of the tibial nerve (NS) results in muscle fatigue from both failure of both contractile elements and synaptic transmission. Direct muscle stimulation (MS) was superimposed on nerve stimulation every 15 s to determine the extent of just contractile element failure. The difference seen in force production between nerve and direct muscle stimulation is the result of failure of synaptic transmission (% Neuromuscular Failure). The picture in Figure 5B shows a typical result in which the direct muscle stimulus (MS) induces responses that drop to a fairly constant value while those induced *via* nerve stimulus (NS) fall to a greater extent. See the Section “Materials and methods” for more details.

The initial ratio of the responses evoked by nerve stimulation vs. those evoked by direct stimulation was 108.2 ± 4.1% for non-crushed soleus prior to the onset of repetitive stimulation. The initial ratio was 93.7 ± 2.9% for AP5&CNQX-blocked and 94.1 ± 3.1 for saline-treated soleus on the side of crush. The ability of the tibial nerve to initially activate the soleus after nerve crush remained impaired compared to the non-crush side at 21 days (*n* = 4 for saline-crush and drug-crush, one-way ANOVA with Tukey *post-hoc* test, *p* = 0.015). No difference, however, was seen in the ratio of the responses to nerve stimulation vs. direct muscle stimulation of the AP5&CNQX -blocked and saline-treated groups (Table 1), indicating that chronic glutamate receptor blockade did not alter the ability of the tibial nerve to stimulate soleus contraction when the NMJ is in a resting state.

As expected, repeated tibial nerve stimulation at 80 Hz resulted in significant neurotransmission failure over time for all three experimental groups (*n* = 4–6 muscles, two-way ANOVA with Tukey *post-hoc* test; *p* < 0.001; Figure 5C). The AP5&CNQX blockade group exhibited less neurotransmission failure beginning at 255 s (Figure 5C). This result suggests that neural transmission failure may be inversely related to the number inputs innervating each muscle fiber. Neuromuscular failure peaked at 85 vs. 69% for saline vs. AP5&CNQX treated mice, a ratio of 1.23. The number of inputs per muscle fiber was 1.2 vs. 1.5 for saline vs. AP5&CNQX treated mice, a ratio of 0.8.

## Discussion

The goal of this study was to assess the possible contribution of NMDA receptors to recovery from denervation of the mouse muscle after sciatic nerve crush. The results of multiple modes of testing indicate that those receptors play a significant role in promoting recovery after sciatic nerve crush. The hypothesis that blocking NMDA receptors would delay recovery was based on our previous evidence for such a role during the first 3 postnatal weeks development of neuromuscular connections. With minor exceptions, discussed below, the results in adults confirm that the activity of NMDA receptors promotes restoration of function during a 3-week period after nerve crush. In both situations, both developing and regenerating axons go through a period of polyneuronal innervation of the muscles, and the pruning process that brings about mono-innervation of each endplate is significantly slowed when glutamate receptors are blocked. Similarly, immunohistochemical staining reveals the presence of NMDA receptors at the endplate, and calcium imaging shows that exogenous activation of NMDA receptors increases intramuscular calcium levels during a restricted period that correlates with establishment of neuromuscular connections. We extended the investigation in adults by testing recovery of behavioral function and found a clear effect of blocking NMDA receptor activity. Further testing using electrophysiological tests revealed mixed evidence for the role of ionotropic glutamate receptors.

### Immunohistochemistry

To examine where and when the receptors are expressed on the muscle, immunohistochemical staining of the gluN1 receptors was employed. Previous studies (Berger et al., 1995; Grozdanovic and Gossrau, 1998; Mays et al., 2009; Malomouzh et al., 2011; Walder et al., 2013; Colombo and Francolini, 2019) have demonstrated the presence of NMDA receptors in adult skeletal muscles in mammals and lizards, but possible changes in expression of the receptors after nerve damage have not been reported. The results in this study showed that NMDA receptor expression is maintained in muscles after sciatic nerve crush, although the degree of co-localization of these receptors with the ∝-bungarotoxin-stained acetylcholine receptors at the NMJ varied. Particularly at early times after denervation, the degree of expression of the NMDA receptors varied from endplate to endplate in each muscle, ranging from undetectable to well-defined (Berger et al., 1995; Grozdanovic and Gossrau, 1998; Malomouzh et al., 2011; Personius et al., 2016; Figure 1).

### Calcium imaging

We previously used calcium imaging with Fluo-4 to study the effects of NMDA during the early postnatal development of mouse EDL and soleus muscles, and we observed that NMDA application significantly raised calcium levels at P4–7 (the earliest age tested) and P11–14 (Personius et al., 2016) but not at later ages. Hence, we now examined whether adult muscles recapitulate such a window of responsiveness to NMDA after nerve crush. To test the contribution of NMDA receptors to rises in muscle calcium after nerve crush, we again used Fluo-4 to examine the responses of the muscles to bath-applied NMDA. Despite the prolonged presence of NMDA receptors that was revealed by immunostaining, NMDA elicited a significant change in muscle calcium levels only for a restricted period, within about 2 weeks after nerve crush. This is a time period when the crushed nerves have reached the muscles and polyneuronal innervation is common (Bauder and Ferguson, 2012).

Thus, the anatomical presence of the receptors does not predict the observable changes in calcium fluxes. The factor that permits this physiological response only during the early period of initial innervation or reinnervation is not yet known. The explanation may lie in changes of the buffering capabilities of the muscles. During development, the early postnatal muscles may be less able to buffer calcium than somewhat older muscles because of the small overall volume of the immature tissue. Similarly, it is possible that the short window after nerve crush when we detect increases in calcium in response to NMDA reflects relatively low calcium buffering capacity during that period.

In these experiments, we did not employ NMDA receptor blockers. In a previous parallel set of experiments on postnatal maturation of the NMJ, we did use the blocker AP5 to assess the specificity of the responses to NMDA, and we showed that the calcium responses were appropriately blocked.

Poor recovery of function after peripheral nerve damage is a prominent clinical phenomenon and is especially associated with situations in which there is a long delay between the time of denervation and the time in which the nerves regain access to the muscle. This relationship between delay time and failure of functional restoration has been elegantly demonstrated experimentally (Sakuma et al., 2016), and we speculate that the requirement that the nerve reach the muscle within a relatively narrow window of time for good functional recovery may be related to the same time window in which activation of the NMDA receptor can elicit muscle calcium responses.

### Polyneuronal innervation

To test whether NMDA receptors promote the elimination of excess innervation after nerve damage in adult mice as they do during normal development (Personius et al., 2016), we examined the innervation of NMJs of normal muscle and of saline-treated vs. APV/CNQX-treated muscles after nerve crush in adults. The muscles were removed and immunostained to reveal regenerating axons and neuromuscular junctions at 14–16 and 21 days post-crush, stages when the process of pruning excess innervation is expected to be partially complete, allowing changes due to receptor blockade to be observable. The APV/CNQX-treated muscles showed significantly slower removal of excess innervation at both stages. These results are consistent with NMDA receptors being necessary for restoration of normal patterns of innervation.

In addition, nerve sprouts that extended beyond the endplate region were seen in the muscles where NMDA receptors had been blocked. Such sprouts are typically associated with extensions of terminal Schwann cell processes, which can act as guides for ingrowing axons (Kang et al., 2019). Thus, it appears that blocking NMDA receptors influences the behavior of terminal Schwann cells.

It is possible that the inclusion of CNQX in the Elvax introduced another variable into these experiments. This blocker was employed to help assure that the NMDA receptors were less likely to open due to glutamate-induced depolarization mediated by AMPA receptors. Because we did not test the effects of APV alone, we cannot conclude that the alteration of synapse elimination was solely due to blocking NMDA receptors. Both the observation of slowed pruning and of unusual nerve sprouts are reminiscent of the effects of acute application of APV to the *Xenopus* tadpole tectum, where blocking NMDA receptors increases optic axon branch turnover (Rajan et al., 1999).

### Behavior

The prolonged presence of polyneuronal innervation at the NMJ after sciatic nerve damage has been implicated in the failure of recovery of motor function (Guntinas-Lichius et al., 2005; Angelov et al., 2007; Skouras et al., 2011). This correlation would imply that AP5&CNQX-block, by prolonging polyneuronal connections at the NMJ, would also interfere with recovery of motor function. In order to assess this possibility, the toe-spread reflex was assessed for a 3-week period after nerve crush with or without inhibitor-infused Elvax (Figure 4). There was initially no difference in the degree of recovery of saline-treated vs. AP5&CNQX-treated mice, but the responses diverged thereafter, with significantly poorer function in the AP5&CNQX-treated limbs by 15 days. This was also essentially the same time at which we saw excess polyneuronal innervation, which continued to at least 21 days post-crush.

The period of 14–16 days post-crush was also when NMDA induced significantly higher calcium responses when compared with saline application. This correlation opens the possibility that there is a discrete window of time when NMDA receptor function is particularly important for restoration of function.

### Muscle mass and force

To test whether the force exerted by the soleus muscles was altered by denervation and/or NMDA receptor blockade, force was examined at different stimulation frequencies. As expected, denervation reduced the force exerted by the muscles (see Figure 5A). However, there was no difference between the force curves of the nerve-crush muscles treated with AP5&CNQX vs. saline. Thus, it seemed likely that there would also be no significant difference between the masses of these denervated sets of muscles and that both groups of denervated muscles would be smaller in mass than normally innervated muscles. Surprisingly, this prediction was only partially fulfilled (Table 1). The crush-saline and non-crush muscles were statistically indistinguishable in mass, and only the crush-drug muscles were reduced in mass. Thus, the AP5&CNQX-treated muscle produces more force per mass than the non-crushed soleus. This paradoxical result may be explicable if there is a change in the composition of the soleus muscles, which normally contain predominantly slow twitch fibers (McDonagh et al., 1980). If the crush-drug muscles’ composition shifts toward a higher percentage of fast twitch fibers than non-crush muscle, then the same mass of muscle would be expected to produce greater force due to the ability of fast twitch fibers to generate higher force than slow twitch fibers.

This conjecture is consistent with our observation that the APV&CNQX-treated muscles trended toward faster time-to-peak twitch force and ½ relaxation times (Table 1), suggesting that AP5&CNQX-treatment may lead to a higher percentage of fast twitch fibers than non-crush muscle, thus producing more force per weight than control.

### Neuromuscular transmission failure

In order to evaluate whether NMDA receptor blockade influenced neurotransmission during repetitive stimulation, we compared the responses of the muscles to direct vs. nerve-evoked repetitive stimulation over a period of 285 s at 21 days post-crush. During most of the stimulation period, the curves of the non-crush, saline-crush, and drug-crush muscle responses were statistically indistinguishable, but during the last 30 s, the APV&CNQX-treated muscles exhibited significantly less neuromuscular failure. It should be noted that the Elvax had been removed from the muscles prior to testing, so the neurotransmission in this work would reflect the state of the muscles when removed from the limb after 21 days of treatment. Presumably, the muscles no longer would contain residual APV or CNQX, and the neurotransmission data shown in Figure 5C would not be influenced by the presence of inhibitors during the recording process.

The continued presence of polyinnervation at the NMJ may underlie the reduced neural transmission failure seen in APV&CNQX-treated muscles. Indeed our data on the number of inputs per muscle fiber suggest that neural transmission failure is inversely related to input number. Polyinnervation at the NMJ results in differential activity-dependent acetylcholine release at the synaptic cleft between relatively active vs. less-active inputs. Following stimulation, weaker inputs are less likely to release acetylcholine than strong inputs (Kopp et al., 2000; Nadal et al., 2016). Thus, polyinnervation itself may reduce neurotransmission failure at the level of the whole muscle by inhibiting ACh release from weaker inputs until the stronger inputs have failed. Indeed, we have previously seen a trend toward reduced neural transmission failure in another system with polyinnervation, namely the diaphragm muscle of mdx mice (an animal model of Duchenne muscular dystrophy) (Personius and Sawyer, 2006). The NMJ of mdx mice demonstrates polyinnervation and axon sprouting similar to that seen in APV&CNQX-treated muscles (Personius and Sawyer, 2005).

### Comparison with development

A motivation for this study was the concept that the regenerating adult neuromuscular system recapitulates aspects of development. Our focus was on the role of the NMDA receptors. The data about the role of NMDA receptors during regeneration in this paper strongly parallels the evidence about NMDA receptors during development. In both cases, we found a limited window of time during which bath-applied NMDA could elicit significant rises in muscle calcium levels, notably at the endplate. Similarly, chronic blockade of glutamate receptors with AP5&CNQX-infused Elvax implants significantly delayed the elimination of supernumerary inputs to each neuromuscular junction.

In our experiments on the role of ionotropic glutamate receptors at the developing NMJ, we concluded that the effects of the AP5&CNQX-infused Elvax implants were primarily due to the blocking of the NMDA receptors rather than to the blocking of the AMPA receptors. This conclusion was based on experiments in postnatal mice that reduced expression of NMDA receptors by use of a 25 base vivo-morpholino that was designed to knock down expression of the GluN1 subunit; these knock-down experiments resulted in the same magnitude of change in polyinnervation that was produced when AP5 and CNQX were infused together *via* Elvax. Similarly, calcium imaging experiments in which only NMDA was applied showed major effects during periods of high plasticity in both the development and regeneration paradigms. Thus, we conclude that during sciatic nerve regeneration in adults, the effect of using AP5&CNQX-infused Elvax are likely ascribed to blocking of the NMDA receptors specifically.

### Contribution of Schwann cells

We have not specifically examined the possible contributions of Schwann cells to the phenomena described in this study. Rodent Schwann cells have been shown to have NMDA receptors (Fink et al., 1999; Liu and Bennett, 2003; Mantuano et al., 2015; Campana et al., 2017). Activation *via* glutamate released from axons mediates a variety of signaling pathways in the Schwann cells but no evidence yet exists of such pathways on the transmission of impulses along the motor axons.

### Possible involvement of NMDA receptors in regulation of neuromuscular transmitter release and synapse stability

There is evidence that NMDA receptor activation can reduce release of acetylcholine at the NMJ of the rat diaphragm (Koyuncuoglu et al., 1998). The mechanism underlying this reduction may be a cascade of events beginning with the activity-related release NAAG from the nerve terminal (Neale and Malomouzh, 2000; Walder et al., 2013), cleavage of NAAG to produce glutamate (Malomouzh et al., 2005; Marmiroli et al., 2012), binding of the glutamate to the postsynaptic NMDA receptors, entry of calcium into the muscle, activation of synaptic nitric oxide synthase, production of nitric oxide, retrograde diffusion of the nitric oxide, and consequent reduction in non-quantal release of acetylcholine (Malomouzh et al., 2003). There is a report that non-quantal release of ACh accelerates elimination of polyneuronal innervation during development (Vyskocil and Vrbova, 1993).

In the central nervous system, the subunit composition of NMDA receptors and their localization at synaptic vs. extrasynaptic sites may play an important role in determining whether the receptors trigger positive or negative influences on the presynaptic cells, respectively (Hardingham et al., 2002; Vanhoutte and Bading, 2003; Lau and Zukin, 2007). Our immunohistochemical results suggest that muscle NMDA receptors are more diffusely distributed during the early stages of regeneration than in the later stages. The subunit composition of the receptors during regeneration has yet to be investigated.

### Other modulators of synapse elimination

Many mechanisms have been implicated in the process of synapse elimination. Some are positive regulators that help to maintain the “winning” synaptic input to the neuromuscular junction, while other are negative regulators that lead to the withdrawal of the other synapses. Consistent with the observations that neuromuscular activity governs synapse elimination (Barry and Ribchester, 1995), many of these proposed regulators are synthesized and/or released in response to activity. For example, presynaptic muscarinic receptors and adenosine receptors as well the brain-derived neurotrophic factor (BDNF) receptors TrkB and p75 all have been implicated in modulating ACh release and influencing pruning [reviewed in Tomàs et al. (2017)].

Another trophic factor that is released in response to neuromuscular stimulation is GDNF (glial-derived neurotrophic factor) (Willand et al., 2016). Overexpression of GDNF in skeletal muscles promotes retention of multiple inputs to developing muscles and to adult muscles after nerve regeneration (Nguyen et al., 1998; Zwick et al., 2001; Gillingwater et al., 2004).

Other components of the NMJ play roles in synapse elimination. MHC1 (Major histocompatibility complex class I) is needed for pruning (Tetruashvily et al., 2016) as are Nfasc155 (the glial isoform of neurofascin) (Roche et al., 2014) and NCAM (neural cell adhesion molecule) (Rafuse et al., 2000).

## Conclusion

These results support the hypothesis that NMDA receptors at the neuromuscular junction play a significant role in re-establishing functional connections after sciatic nerve crush in adult mice. In both this and our previous studies of normal development, there appears to be a restricted period of time during which the receptors influence the transition from polyneuronal innervation to single innervation. This window of time corresponds to the period when application of NMDA produces a rise the muscle calcium levels. In addition, these data in adult mice demonstrate significant effects of NMDA receptor activation on preventing muscle atrophy and promoting motor recovery.

## Data availability statement

The raw data supporting the conclusions of this article will be made available by the authors, without undue reservation.

## Ethics statement

This animal study was reviewed and approved by Institutional Animal Care and Use Committee of the University at Buffalo.

## Author contributions

KP and SU conducted a review of the literature and wrote the manuscript. KP performed electrophysiological, imaging, and behavioral experiments. DS assisted with data analysis. DK performed electrophysiological experiments. SU produced Elvax implants and performed immunohistochemical and calcium imaging experiments. All authors contributed to the article and approved the submitted version.
